# Patients with hemodialysis-induced hypoxemia had a poor prognosis of COVID-19

**DOI:** 10.1186/s41100-022-00408-5

**Published:** 2022-05-20

**Authors:** Masataro Toda, Ayumi Yoshifuji, Kentaro Fujii, Motoaki Komatsu, Ai Kato, Ikue Tamura, Wataru Sugi, Munekazu Ryuzaki

**Affiliations:** Department of Nephrology, Tokyo Saiseikai Central Hospital, 1-4-17 Mita, Minato-ku, Tokyo, 108-0073 Japan

**Keywords:** COVID-19, Hemodialysis, Hypoxemia, Cytokine storm

## Abstract

**Background:**

We experienced that some hemodialysis (HD) patients with coronavirus disease 2019 (COVID-19) exacerbated hypoxemia during HD. Though HD-induced hypoxemia has been reported, there have been no reports of HD-induced hypoxemia in patients with COVID-19 and its effect on prognosis of COVID-19.

**Methods:**

Eleven HD patients admitted with COVID-19 from August 2020 to April 2021 were classified into the patients whose oxygen demand increased by more than 3 L/min with mask during HD (worsened group, *n* = 5) and others (not-worsened group, *n* = 6). The background, laboratory findings, severity of COVID-19 and prognosis were compared between the two groups. In addition, blood gases were measured before and after dialysis among HD patients admitted with COVID-19 on April 2021 (*n* = 3).

**Results:**

There were no significant differences in backgrounds, except for a higher proportion of diabetes mellitus in worsened group (*p* = 0.04). Although laboratory findings were not significantly different on admission day, albumin and LDH levels 7 days after admission were significantly lower and higher in worsened group, respectively (*p* = 0.03 and < 0.01). The severity of COVID-19 and survival rate were significantly worse in worsened group (*p* = 0.01 and 0.03). The alveolar-arterial oxygen pressure difference (Aa-DO_2_) opened during HD in a patient with HD-induced hypoxemia, but did not open in patients without HD-induced hypoxemia.

**Conclusions:**

There is a close relationship among HD-induced hypoxemia and poor prognosis of COVID-19. The HD-induced hypoxemia of patients with COVID-19 may be caused by ventilation/perfusion mismatching.

**Supplementary Information:**

The online version contains supplementary material available at 10.1186/s41100-022-00408-5.

## Background

Since the first case of coronavirus disease 2019 (COVID-19) in December 2019, more than hundreds of million people infected COVID-19 worldwide. Hemodialysis (HD) patients are at a much higher risk of COVID-19 than the general population and have nearly 20% mortality rate of COVID-19 (1). This is considered to be attributed to dysfunction of both innate and adaptive immune systems and number of comorbid underlying diseases of HD patients (2). Among HD patients with COVID-19, older age, male, longer duration of dialysis, diabetic mellitus (DM), and cardiovascular disease were identified as predictors of poor prognosis (3). However, it is unclear whether HD itself affects severity and prognosis of HD patients with COVID-19.

As the clinical symptoms of COVID-19 rapidly become severe due to “cytokine storm,” which means a systemic high inflammation caused by uncontrolled overproduction of cytokines, immunomodulators including steroids and various cytokine receptor inhibitors have been used to suppress cytokine storm in patients with COVID-19 (4). Generally, dialysis activates various immune pathways, such as complement cascade, neutrophils, monocytes, inducing systemic inflammation (2). Since HD patients with COVID-19 also showed increased cytokine production after dialysis compared to before, especially in critical cases (5), HD itself may provoke systemic inflammation, worsening COVID-19 prognosis.

We experienced that some HD patients hospitalized with COVID-19 had exacerbation of respiratory condition during HD. While some HD-induced hypoxemia in non-COVID-19 patients has been reported (6, 7), there are no reports on patients with COVID-19. To best of our knowledge, this is the first study to examine the mechanism why some patients with COVID-19 worsened their respiratory condition during HD and its effect on prognosis.

## Methods

Eleven HD patients admitted to our hospital with COVID-19 from August 2020 to April 2021. Before February 2021, all patients were treated with standard therapy (dexamethasone and antiviral therapy). After February 2021, patients who underwent high-flow nasal cannula (HFNC) oxygen therapy were treated with tocilizumab (TCZ) (8 mg/kg) and methylprednisolone (mPSL) (500 mg/day [≤ 75 kg], 1000 mg/day [> 75 kg]) for 3 days in addition to standard therapy (Additional file 1: Table S1). We classified them into the patients whose oxygen demand increased by more than 3 L/min with mask during HD (worsened group) (*n* = 5) and patients whose oxygen demand did not increase during HD (not-worsened group) (*n* = 6). The background, laboratory findings (albumin, CRP, LDH, ferritin, D-dimer and lymphocyte count), severity of COVID-19 and prognosis were compared. The severity of COVID-19 was categorized according to the following condition; “Severe” as a case with a SpO_2_ less than 94% on room air at sea level and required oxygen therapy; “Critical” as a case requiring HFNC or intubation (patients were intubated when 10 L/min of oxygen was required to maintain SpO_2_ above 94%, but if intubation was not consented, they were placed on HFNC). Changes in oxygen demand and LDH were evaluated after hospitalization in five patients of worsened group (CRP was strongly suppressed by TCZ, and an inadequate indicator of COVID-19 pneumonia severity, therefore LDH was used as a marker of COVID-19 severity and lung injury (8)). Furthermore, blood gases were measured before and after dialysis in patients admitted on April 2021 (*n* = 3), as an additional study.

Median values were compared using the Mann–Whitney *U* test. Frequencies between groups were compared using Fisher’s exact test or the Chi-square test. Statistical significance was set at *p* < 0.05. Statistical analysis was performed using GraphPad Prism 9.

## Results

### Clinical course of patients whose oxygen demand increased during dialysis

**Fig. 1:**
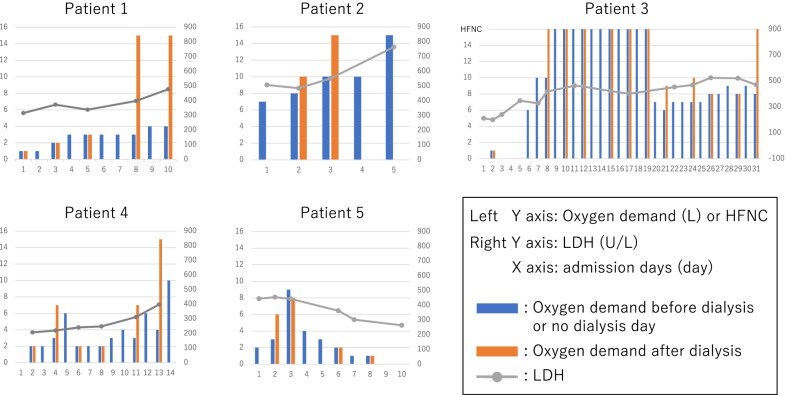
Clinical course of patients whose oxygen demand increased during dialysis. Five patients whose oxygen demand increased by more than 3 L/min with mask during post-hospitalization dialysis. Patient 1–4 were intubated or placed on HFNC after their oxygen demand greatly increased during dialysis. Among these four patients, LDH levels tended to increase with elevated oxygen demand. Patient 5 whose oxygen demand once increased during dialysis improved his condition and no longer needed the oxygen demand with decrease trend of LDH levels. HFNC; high-flow nasal cannula, LDH; lactate dehydrogenase

Figure 1 showed the clinical course of 5 patients whose oxygen demand increased by more than 3 L/min with mask during post-hospitalization dialysis. Patient 1 whose oxygen demand greatly increased during dialysis on day 8 after admission placed on HFNC on day 11 and then, he died 32 days later. Patient 2 whose oxygen demand increased during dialysis on day 3 after admission was intubated on day 6 and died one day later. Patient 3 whose oxygen demand increased during dialysis day 8 after admission was placed on HFNC on the same day. His respiratory condition improved, and he was weaned HFNC off on day 20. However, his oxygen demand increased during dialysis on day 21 and 24, so he was placed on HFNC again after dialysis on day 31 and then, he died two days later. Patient 4 whose oxygen demand greatly increased during dialysis day 13 after admission was placed on HFNC on day 15. His respiratory condition improved, and he was weaned HFNC off on day 22 with a favorable clinical outcome. Among these four patients, LDH levels tended to increase with elevated oxygen demand. Patient 5 whose oxygen demand increased during dialysis on day 2 after admission improved his condition and no longer needed the oxygen demand with decrease trend of LDH levels (Fig. 1).

### Comparisons between worsened group and not-worsened group

**Fig. 2:**
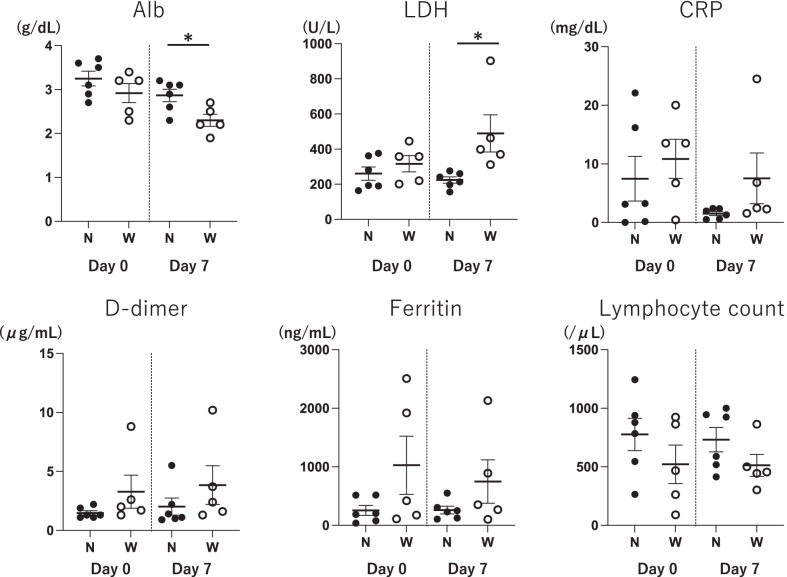
Between-group comparisons of laboratory data. There were no significant differences in laboratory findings on Day 0. However, albumin and LDH levels on Day 7 were significantly lower and higher in worsened group than in not-worsened group, respectively (*p* = 0.03 and < 0.01). In addition, CRP, ferritin and D-dimer levels were tended to be higher and lymphocyte count level was tended to be lower in worsened group on Day 7. N; not-worsened group, W; worsened group, Alb; albumin, LDH; lactate dehydrogenase, CRP; C-reactive protein, Day 0; admission day, Day 7; 7 days after admission

As the backgrounds, there were no significant differences in age, sex, body mass index, dialysis vintage, causes of end-stage renal disease and the presence of cancer between two groups. However, the proportion of DM in the worsened group was significantly higher than in the non-worsened group (*p* = 0.04) (Table 1). Although no significant differences were observed in laboratory findings on admission day, albumin and LDH levels 7 days after admission were significantly lower and higher in worsened group than in not-worsened group, respectively (*p* = 0.03 and < 0.01). In addition, day 7 after admission, CRP, ferritin and D-dimer levels tended to be higher and lymphocyte count level tended to be lower in worsened group (Table 1, Fig. 2). In worsened group, 4 out of 5 patients demanded 10 L/min of oxygen with mask to maintain SpO_2_ above 94% and were placed on HFNC or intubation, while no one in not-worsened group (*p* = 0.01). Therefore, the severity of COVID-19 is significantly worse in the worsened group than in not-worsened group (*p* = 0.01). Further, worsened group had significantly lower survival rate than not-worsened group (*p* = 0.03) (Table 1).

**Table 1:** Backgrounds, laboratory data, outcome of patients hospitalized with COVID-19

			Worsened (*n* = 5)	Not-worsened (*n* = 6)	*p* value
Backgrounds	Age (y)		71.8	61.8	0.173
	Male n (%)		3 (60)	4 (67)	> 0.999
	BMI (kg/m^2^)		23.0	24.5	0.716
	Dialysis vintage (y)		6.2	7.7	0.646
	Causes of ESRD n (%)	DM	4 (80)	1 (17)	0.103
		Sclerosis	1 (20)	4 (67)	
		MN	0 (0)	1 (17)	
	Smoke n (%)		2 (40)	4 (67)	0.567
	Comorbidity n (%)	DM	4 (80)	1 (17)	**0.036**
		Hypertension	3 (60)	6 (100)	0.182
		Hyperlipidemia	0 (0)	3 (50)	0.182
		Cardiovascular disease	3 (60)	3 (50)	> 0.999
		Lung disease	1 (20)	0 (0)	0.455
		Malignant tumor	2 (40)	1 (17)	0.546
Laboratory data on admission (min-max)	Alb (g/dL)		2.92 (2.3–3.4)	3.25 (2.7–3.7)	0.305
	LDH (U/L)		316.4 (201–445)	261.0 (164–376)	0.398
	CRP (mg/dL)		10.85 (0.43–20.03)	7.47 (0.00–22.10)	0.502
	Ferritin (ng/mL)		1027.8 (112–2508)	256.3 (38–518)	0.429
	Lymphocyte count (/μL)		521.9 (91–924)	776.3 (265–1245)	0.247
	D-dimer (μg/mL)		3.28 (1.3–8.8)	1.48 (1.1–2.2)	0.113
Laboratory data 7 days after admission (min-max)	Alb (g/dL)		2.3 (1.9–2.7)	2.9 (2.3–3.2)	**0.026**
	LDH (U/L)		489.6 (312–903)	224.2 (156–276)	**0.004**
	CRP (mg/dL)		7.53 (2.28–24.5)	1.52 (0.51–2.39)	0.082
	Ferritin (ng/mL)		749.6 (104–2131)	262.0 (105–552)	0.329
	Lymphocyte count (/μL)		513.5 (304–864)	732.2 (414–1001)	0.126
	D-dimer (μg/mL)		3.8 (1.3–10.2)	2.0 (0.9–5.5)	0.178
Outcome n (%)	Treatment complications		2 (40)	2 (33)	> 0.999
	Severity	Severe	1 (20)	6 (100)	**0.006**
		Critical	4 (80)	0 (0)	
	HFNC, Intubate		4 (80)	0 (0)	**0.006**
	Survive		2 (40)	6 (100)	**0.026**

Bold values indicate* p*-value < 0.05

COVID-19; coronavirus disease 2019, BMI; body mass index, CKD; chronic kidney disease, DM; diabetes mellitus, MN; membranous nephropathy, Alb; albumin, LDH; lactate dehydrogenase, CRP; C-reactive protein, HFNC; high-flow nasal cannula

### Changes in blood gases before and after dialysis

Among three patients, blood gases were measured before and after dialysis, only one patient whose oxygen demand with mask increased more than 3 L/min and PO_2_/FiO_2_ ratio worsened from 359.4 to 169.6 mmHg during dialysis. The blood gas findings showed that the alveolar-arterial oxygen pressure difference (Aa-DO_2_) opened from 79.2 to 227.3 in a patient with oxygen demand increased, but the Aa-DO_2_ almost did not change in other two patient whose oxygen demand did not increase. The pH increased to alkalosis, but there was no pCO_2_ retention in all patients (Table 2).

**Table 2:** Blood gases before and after dialysis in patients with COVID-19

	Worsened		Not-worsened			
	Case 1		Case 2		Case 3	
	Before	After	Before	After	Before	After
Blood pressure (mmHg)	161/71	146/84	151/69	162/67	106/63	117/70
Oxygen (L) or FiO_2_	3	6	4	4	55%	55%
pH	7.419	7.578	7.389	7.551	7.400	7.522
pO_2_ (mmHg)	115.0	84.8	79.8	57.7	74.0	74.1
pCO_2_ (mmHg)	32.9	35.5	32.9	31.5	36.0	35.0
P/F ratio	359.4	169.6	215.7	155.9	134.5	134.7
HCO_3_ (mEq/L)	20.8	32.4	19.4	27.0	21.8	28.1
Aa-DO_2_ (mmHg)	79.2	227.3	142.9	166.7	273.2	274.3

COVID-19; coronavirus disease 2019, P/F ratio; PO_2_/FiO_2_ ratio, Aa-DO_2_; alveolar-arterial oxygen pressure difference

## Discussion

In this study, we experienced that respiratory condition worsened during HD in patients with severe or critical COVID-19, and clarified the close relationship between HD-induced hypoxemia and poor prognosis of COVID-19.

Our data demonstrated that the worsened group had a significantly higher severity and required HFNC more frequently than the not-worsened group (Fig. 1, Table 1). We hypothesized that two processes may be involved in the mechanism by which HD hypoxemia is more likely to occur in severe to critical COVID-19 patients. Firstly, the dialysis membranes induce recruitment of neutrophils and monocytes, which release pro-inflammatory cytokines, such as interleukin 1, interleukin 6 (IL-6), and tumor necrosis factor-α. In fact, IL-6 after HD was reported to be elevated compared to that before HD in COVID-19 patients, especially in critical cases (5). Secondly, HD activates complement cascade both alternative pathway and lectin pathway due to absorbing inhibitors of these pathways by dialysis membranes (2). Through these processes, dialysis membranes and activated complement recruit neutrophils and monocytes into blood vessels, and then, these cells accumulate and occlude the pulmonary vascular bed, causing ventilation/perfusion mismatching, leading to HD-induced hypoxemia. In addition, the ventilation/perfusion mismatching is also considered to be caused by lung interstitial edema resulted from increased pulmonary vascular permeability due to activated complement and neutrophilic adhesion. Furthermore, in severe COVID-19, the prevalence of alveolar-capillary microthrombi is nine times higher than in influenza. In COVID-19, D-dimer is reported as the most useful marker for thrombosis (9), and the worsened group tended to have higher D-dimer levels on admission and 7 days after admission than the not-worsened group. One of the mechanisms of this is platelet activation by activated neutrophils via neutrophil extracellular traps (NETs) and platelet-monocyte aggregates forming microthrombi. Also, the induction of endothelial adhesion molecules, neutrophils, monocytes and platelet activation by complement activation is related above mechanism (10, 11). Therefore, the induction of neutrophils, monocytes and complement activation by dialysis may cause pulmonary vascular microthrombosis resulting in the ventilation/perfusion mismatching. According to our results of blood gases before and after dialysis, the Aa-DO_2_ opened from 79.2 to 227.3 in a patient with oxygen demand increased, but the Aa-DO_2_ almost did not change in patients whose oxygen demand did not increase, suggesting that the ventilation/perfusion mismatching causes HD-induced hypoxemia. In addition, there was a significantly higher proportion of DM patients in the worsened group compared with the not-worsened group. It was reported that IL-6 after HD was elevated in DM patients compared to before HD (12, 13). It could be assumed that COVID-19 patients with DM were more prone to inflammation due to dialysis, contributing to ventilation/perfusion mismatching. On the other hand, the other causes of HD-induced hypoxemia include hypoventilation-induced hypoxemia due to increase in pH by bicarbonate influx during HD, sleep apnea when they sleep during HD, decreased cardiac output due to decreased blood volume, and exacerbation of cardiogenic pulmonary edema by supine position (7) have been reported. However, our study shows pH increased, but pCO_2_ did not retain in both patients with and without increased oxygen demand. In addition, there is no patient who slept or markedly decreased blood pressure during HD or had cardiac failure. These facts indicate that the HD-induced hypoxemia of patients with COVID-19 may be caused by ventilation/perfusion mismatching.

COVID-19 causes the excessive immune response to SARS-CoV-2 in severe to critical condition, which is called cytokine storm, leading to multiple organ failure including impaired respiratory function. (4). In our study, patients with HD-induced hypoxemia (worsened group) had significantly worse severity of COVID-19 and lower survival rates compared to not-worsened group (Table 1, Fig. 2). Therefore, hypoxemia during dialysis may be a predictor of poor prognosis in HD patients with COVID-19. It has been reported that the induction of inflammation by HD is a poor prognostic factor of COVID-19 (5), suggesting that the hypoxemia as a clinical outcome of HD-induced inflammation may be considered a poor prognostic factor as well. Furthermore, in COVID-19, LDH levels are positively associated with systemic inflammation (8), and high LDH and low albumin have been reported as predictors of poor prognosis in HD patients with COVID-19. In this study, the worsened group had significantly higher LDH and lower albumin levels than the not-worsened group on day 7 of admission, even though there was no significant difference at admission (14) (15). Although it is not clear whether patients with HD-induced hypoxia are more likely to develop critical condition or patients with critical COVID-19 develop HD-induced hypoxia, our results suggest that there is a close relationship between HD-induced hypoxia and the poor prognosis of COVID-19.

Important limitations of our study include the small number of patients. In addition, we could not investigate the change of inflammatory markers such as IL-6 and activated complement during dialysis.

## Conclusion

There is a close relationship among HD-induced hypoxemia and poor prognosis of COVID-19. The HD-induced hypoxemia of patients with COVID-19 may be caused by ventilation/perfusion mismatching.

## Supplementary Information

**Additional file 1**.** Supplement Table 1**. Clinical information of all patients hospitalized with COVID-19.

## Data Availability

The datasets used and/or analyzed during the current study are available from the corresponding author on reasonable request.

## Abbreviations

HD: Hemodialysis
COVID-19: Coronavirus disease 2019
DM: Diabetes mellitus
HFNC: High-flow nasal cannula
Aa-DO_2_: Alveolar-arterial oxygen pressure difference
