# Supplementary material for: Patients with hemodialysis-induced hypoxemia had a poor prognosis of COVID-19
Source: Ren Replace Ther. 2022 May 20;8(1):22. doi: 10.1186/s41100-022-00408-5 (PMC9122251; doi:10.1186/s41100-022-00408-5)
Supplement: Supplementary file 1 — Additional file 1. Supplement Table 1. Clinical information of all patients hospitalized with COVID-19. [file 41100_2022_408_MOESM1_ESM.docx]

| **Background** | **Age (year-old)** | 72 | 73 | 70 | 86 | 58 | 49 | 55 | 58 | 56 | 80 | 73 |
| --- | --- | --- | --- | --- | --- | --- | --- | --- | --- | --- | --- | --- |
|  | **Sex** | M | F | M | F | M | M | M | M | F | F | M |
|  | **BMI (kg/m^2^)** | 22.6 | 24.0 | 24.7 | 23.2 | 20.2 | 36.3 | 23.0 | 34.7 | 12.3 | 22.2 | 18.7 |
|  | **Dialysis vintage (y)** | 16 | 1 | 4 | 2 | 9 | 13 | 8 | 23 | 4 | 5 | 3 |
|  | **Causes of CKD** | DM | DM | DM | DM | Sclerosis | Sclerosis | MN | Sclerosis | Sclerosis | Sclerosis | DM |
| **Comorbidity** | **Diabetes** | ○ | ○ | ○ | ○ | × | × | × | × | × | × | ○ |
|  | **Hypertension** | × | ○ | ○ | × | ○ | ○ | ○ | ○ | ○ | ○ | ○ |
|  | **Hyperlipidemia** | × | × | × | × | × | ○ | ○ | × | × | × | ○ |
|  | **Malignant tumor** | × | ○ | × | ○ | × | × | × | × | × | ○ | × |
|  | **Cardiovascular disease** | ○ | ○ | ○ | × | × | ○ | × | ○ | × | ○ | × |
|  | **Lung disease** | × | × | × | ○ | × | × | × | × | × | × | × |
|  | **Smoke** | ○ | ○ | × | × | × | × | ○ | ○ | ○ | ○ | × |
| **Treatment** | | FPV DEX | FPV DEX TCZ P | FPV DEX | FPV DEX TCZ P | FPV DEX | FPV DEX | FPV DEX | FPV DEX | FPV DEX | FPV DEX | FPV DEX TCZ P |
| **Treatment complications** | | No | Sepsis | No | Sepsis | No | Sepsis | No | No | No | UTI, Cerebellar hemorrhage | No |
| **COVID-19** | **Severity** | Critical | Critical | Critical | Critical | Severe | Severe | Severe | Severe | Severe | Severe | Severe |
|  | **Admission days** | 48 | 26 | 18 | 34 | 38 | 25 | 11 | 23 | 21 | 39 | 27 |
|  | **Outcome** | death | discharge | death | death | discharge | discharge | discharge | discharge | discharge | transfer | discharge |
| **Laboratory data　　　 on admission** | **Alb (g/dL)** | 3.4 | 3.2 | 3.2 | 2.5 | 2.3 | 3.1 | 3.7 | 2.9 | 2.7 | 3.5 | 3.6 |
|  | **LDH (U/L)** | 358 | 220 | 358 | 201 | 445 | 281 | 362 | 376 | 193 | 190 | 164 |
|  | **CRP (mg/dL)** | 13.53 | 0.43 | 13.53 | 6.73 | 20.03 | 22.10 | 3.24 | 16.18 | 0.00 | 3.12 | 0.16 |
|  | **Ferritin (ng/mL)** | 176 | 421 | 1922 | 112 | 2508 | 203 | 185 | 518 | 515 | 79 | 38 |
|  | **Lymphocyte count (/μL)** | 864 | 924 | 468 | 91 | 263 | 784 | 546 | 265 | 879 | 939 | 1245 |
|  | **D-dimer (μg/mL)** | 1.3 | 2.0 | 1.7 | 8.8 | 2.6 | 1.9 | 1.1 | 1.3 | 1.1 | 2.2 | 1.3 |
| **Laboratory data　　　 7 days after admission** | **Alb (g/dL)** | 2.7 | 2.5 | 1.9 | 2.2 | 2.2 | 3.1 | 2.9 | 2.6 | 2.3 | 3.1 | 3.2 |
|  | **LDH (U/L)** | 399 | 312 | 903 | 370 | 370 | 260 | 241 | 276 | 156 | 214 | 198 |
|  | **CRP (mg/dL)** | 2.47 | 6.82 | 24.5 | 2.28 | 2.28 | 2.36 | 0.61 | 2.39 | 1.29 | 1.96 | 0.51 |
|  | **Ferritin (ng/mL)** | 104 | 354 | 2131 | 890 | 890 | 251 | 231 | 307 | 552 | 105 | 126 |
|  | **Lymphocyte count (/μL)** | 864 | 500 | 456 | 304 | 444 | 946 | 518 | 414 | 925 | 589 | 1001 |
|  | **D-dimer (μg/mL)** | 1.3 | 3.7 | 2.4 | 10.2 | 1.6 | 2.2 | 1.1 | 0.9 | 1.4 | 5.5 | 1.0 |

**Supplement Table 1. Clinical information of all patients hospitalized with COVID-19**

BMI; body mass index, CKD; chronic kidney disease, DM; diabetes mellitus, MN; membranous nephropathy, COVID-19; coronavirus disease 2019, Alb; albumin, LDH; lactate dehydrogenase, CRP; C-reactive protein, FPV; favipiravir, TCZ; tocilizumab (8 mg/kg), P; methylprednisolone pulse (three days of 500 mg/day and 1000 mg/day for < 75 kg and > 75 kg, respectively), DEX; dexamethasone, UTI; urinary tract infection
